# Disease gene discovery in male infertility: past, present and future

**DOI:** 10.1007/s00439-020-02202-x

**Published:** 2020-07-07

**Authors:** M. J. Xavier, A. Salas-Huetos, M. S. Oud, K. I. Aston, J. A. Veltman

**Affiliations:** 1 Biosciences Institute, Faculty of Medical Sciences, Newcastle University, Newcastle-upon-Tyne, UK; 2 Andrology and IVF Laboratory, Department of Surgery (Urology), University of Utah, Salt Lake City, USA; 3 Department of Human Genetics, Radboud University Medical Centre, Nijmegen, Netherlands

## Abstract

Identifying the genes causing male infertility is important to increase our biological understanding as well as the diagnostic yield and clinical relevance of genetic testing in this disorder. While significant progress has been made in some areas, mainly in our knowledge of the genes underlying rare qualitative sperm defects, the same cannot be said for the genetics of quantitative sperm defects. Technological advances and approaches in genomics are critical for the process of disease gene identification. In this review we highlight the impact of various technological developments on male infertility gene discovery as well as functional validation, going from the past to the present and the future. In particular, we draw attention to the use of unbiased genomics approaches, the development of increasingly relevant functional assays and the importance of large-scale international collaboration to advance disease gene identification in male infertility.

## Introduction

Infertility is a complex pathological condition that affects close to 7% of the global male human population and presents as a wide range of heterogeneous phenotypes, from congenital or acquired urogenital abnormalities, endocrine disturbances and immunological factors to spermatogenic quantitative and qualitative defects (Krausz 2011; Tournaye et al. 2017). While severe forms of infertility cannot be directly inherited—by default an affected man is incapable of naturally passing on his genetic information (unless facilitated by assisted reproductive technologies)—genetics plays a major role in this disorder. Unaffected parents can for example pass on genetic mutations that result in autosomal recessive or X-linked forms of male infertility (Chillón et al. 1995; Yatsenko et al. 2015). In addition, many mutational events take place during normal gametogenesis that can result in germline de novo mutations (DNMs), de novo copy number variations (CNVs) or de novo chromosomal abnormalities, some of which can result in male infertility in the offspring (Jacobs and Strong 1959; Reijo et al. 1995; Sun et al. 1999).

In approximately 15% of infertile men a genetic defect is most likely the underlying cause of the pathology (Tournaye et al. 2017; Krausz and Riera-Escamilla 2018). However, despite widespread usage of karyotyping, azoospermia factor (AZF) deletion screening and cystic fibrosis transmembrane conductance regulator (*CFTR*) mutation analysis, a recent study in a large unselected patient cohort revealed a causal genetic diagnosis in only 4% of infertile men (Tüttelmann et al. 2018). Similar to what has been found for other disorders, genetics is found to play a more prominent role in the most severe forms of spermatogenic impairment such as severe oligozoospermia (< 5 million sperm cells per ml) or azoospermia (no sperm in ejaculate) (Lopes et al. 2013; Krausz and Riera-Escamilla 2018).

**Fig. 1:**
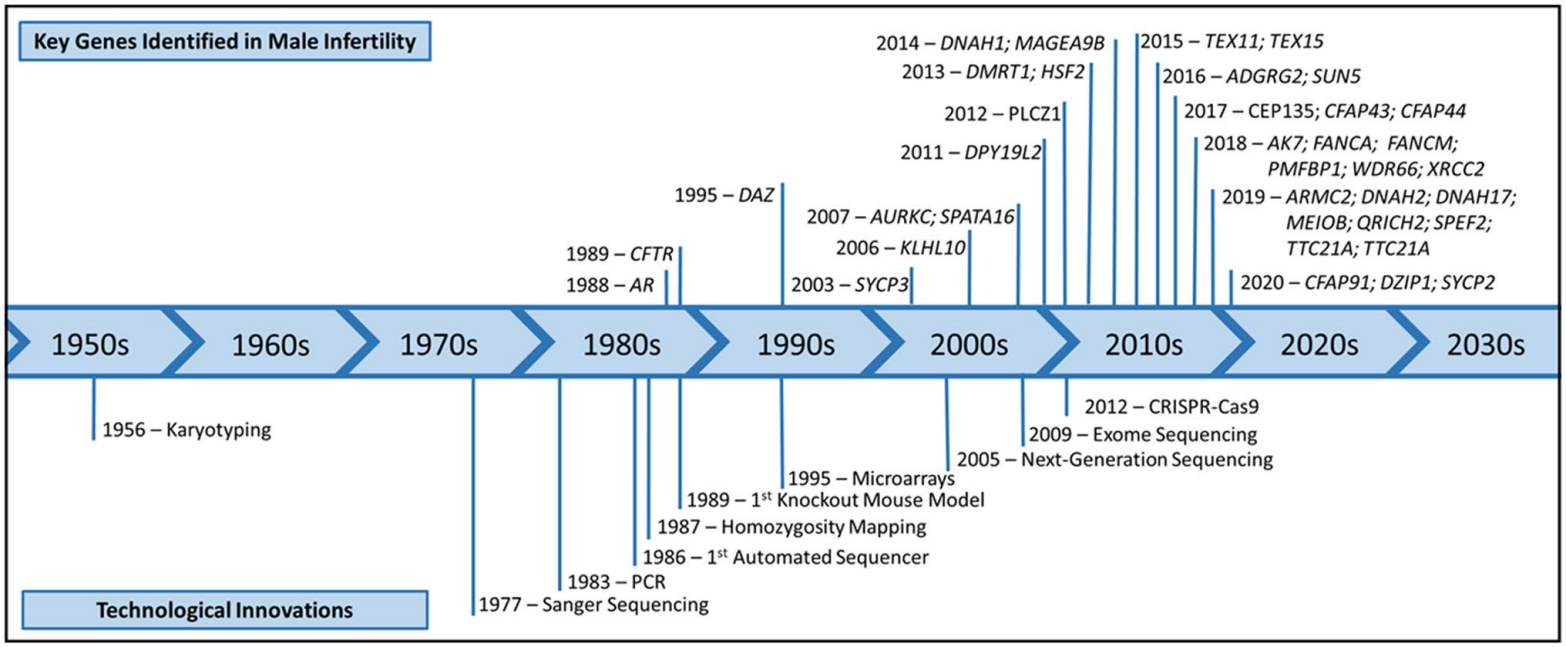
Timeline of the discovery of key genes involved in male infertility. The development of novel molecular techniques and technological advancements introduced since 1956 have allowed for the identification of key genes responsible for the various types of male infertility in humans. Although the first male infertility genes were first identified in the late 1980s, the widespread application of microarray and NGS approaches has resulted in an increase in the detection of male infertility genes

The identification of genetic causes of male infertility, which began in the middle of the twentieth century, continues to this day aided by the development of novel molecular techniques and technological advancements that have allowed for the discovery and characterisation of key genes responsible for the various subtypes of human male infertility (Fig. 1). Although evidence for a genetic cause of male infertility was first reported in the 1950s, when an extra X chromosome was found in patients diagnosed with Klinefelter Syndrome, it was not until the late 1990s that research efforts were intensified to search for additional genetic factors, initially focusing on specific deletions and mutations in the androgen receptor and the *CFTR* gene (Dumur et al. 1990; Akin et al. 1991; McPhaul et al. 1992; Patrizio et al. 1993) and Y chromosomal abnormalities (Vogt et al. 1992; Reijo et al. 1995). In recent years, researchers have increasingly applied array-based genome-wide approaches and, more recently, next-generation sequencing (NGS) technologies to perform more unbiased genomic studies in male infertility and identify numerous male infertility genes (Aston and Carrell 2009; Talkowski et al. 2011; Tüttelmann et al. 2011; Ayhan et al. 2014).

In this review, we contextualise how different technological advances have contributed to our understanding of the genetics of male infertility, detailing current shortcomings and challenges. We conclude by providing some perspective on future directions and a call-to-arms among researchers, clinicians and patients for stronger collaboration and sharing of information for the benefit of all.

## The past: chromosome studies and specific gene analysis revealed the first male infertility genes

### Chromosomal abnormalities causing male infertility

Karyotyping was the first test employed to investigate the presence of genetic abnormalities in infertile men and to this day remains the most widely used diagnostic test in male infertility. This cytogenetic technique revealed several very important chromosomal abnormalities associated to male infertility, with the most common being the presence of an additional X chromosome (47, XXY) which characterises Klinefelter syndrome (Jacobs and Strong 1959), present in 15% of non-obstructive azoospermic patients (Ferlin et al. 2006a; Jungwirth et al. 2012; Punab et al. 2016; Vockel et al. 2019). The combination of karyotype analysis with fluorescence in situ hybridisation (FISH) revealed additional chromosomal abnormalities to underlie primary infertility and sex development disorders, specifically 46, XX male; Robertsonian and reciprocal translocations (Therkelsen 1964; Chapelle et al. 1964; Hamerton 1968; Koulischer and Schoysman 1974; Jacobs et al. 1975).

Karyotype analysis was also essential to identify the location of genetic factors controlling normal spermatogenesis. It was through the usage of this technique that in 1976 a deletion at the distal portion of band q11 of the Y chromosome was found in 6 men with azoospermia and the region was identified as essential for spermatogenesis (Tiepolo and Zuffardi 1976). Although this pointed to the presence of a gene or genes controlling human spermatogenesis in this deleted portion of the Y chromosome, it was not until the 1990s that the first gene directly involved in spermatogenic failure was identified by novel molecular technologies.

The search for the azoospermia factor (AZF) within the deleted regions of the Y chromosome involved the use of polymerase chain reaction (PCR) analysis and Y-specific sequence-tagged sites (STSs) to identify potential candidate genes (Ma et al. 1992, 1993; Kobayashi et al. 1994). Appropriately, the strongest candidate gene found to be absent in the azoospermic men with Y microdeletions was named Deleted in Azoospermia or *DAZ* (Reijo et al. 1995, 1996; Vogt 1998; Ferlin et al. 1999)*,* of which four paralogs (*DAZ1-4*) have now been identified on the Y chromosome (Saxena et al. 2000). Further application of these PCR-based techniques revealed three genomic regions on the Y chromosome frequently deleted in men with spermatogenic failure, named AZF regions a, b, and c (Vogt 1998). Each of these regions contains candidate genes highly or exclusively expressed in the testis and essential in spermatogenesis, including *BPY2, CDY, DAZ, HSFY, RBMY, PRY, TSPY; VCY* and *XKRY* (Reijo et al. 1995; Elliott et al. 1997; Lahn and Page 1997; Yen 1998; Sun et al. 1999; Skaletsky et al. 2003; Krausz and Casamonti 2017). Microdeletions affecting AZF regions result in a variable phenotype, ranging from oligozoospermia to azoospermia in 2–10% in infertile men (Krausz and Riera-Escamilla 2018). However, the genetic causes for the infertility of most men remained unknown, requiring scientists to look elsewhere in the genome.

### Identification of specific gene mutations resulting in male infertility

The first gene linked to male infertility outside of the Y chromosome was identified in 1988 on chromosome X. At the time, mutations in the murine androgen receptor gene, mapped via linkage analysis to the X chromosome, had already been well established to cause testicular feminisation in mice (Lyon and Hawkes 1970). Based on this information, Brown et al*.* used a PCR-based approach coupled with southern blotting to reveal that deletion of the human Androgen Receptor (*AR*, also known as *NR3C4*) was responsible for infertility in patients with mild or partial androgen insensitivity syndrome, as well as sex reversal in patients with complete androgen insensitivity syndrome (Brown et al. 1988). Initially, PCR-amplified exons of the *AR* gene were screened for mutations using techniques such as denaturing gradient gel electrophoresis (DGGE) and single-strand conformational polymorphism analysis (SSCP) and by denaturing high performance liquid chromatography (DHPLC) screening (Quigley et al. 1995; Ferlin et al. 2006b). Since the gene was first linked to male infertility, mutations in *AR* have been identified in 2% of infertile men causing either mild or partial androgen insensitivity syndrome (McPhaul et al. 1992; Ferlin et al. 2006b; Gottlieb et al. 2012; Vockel et al. 2019).

In 1989, mutations in the *CFTR* gene (Cystic Fibrosis Transmembrane Conductance Regulator) on chromosome 7 were discovered to underlie Cystic Fibrosis, using restriction fragment length polymorphism (RFLP) analysis to pinpoint the genomic locus of interest followed by PCR-based sequencing to identify mutations (Kerem et al. 1989; Riordan et al. 1989). Since the original reporting, further studies have identified specific mutations in this gene responsible for isolated infertility. In particular, mutations in *CFTR* have been found to cause obstructive azoospermia as a result of Congenital Bilateral Absence of the Vas Deferens using DGGE and SSCP techniques (Dumur et al. 1990; Anguiano 1992; Culard et al. 1994). The high prevalence of *CFTR* mutations in the global population, particularly in individuals of European descent where 1 in 25 are carriers of pathogenic variants, explains why *CFTR* gene mutations cause 60–70% of congenital absence of the vas deferens (CAVD) cases (Weiske et al. 2000). Overall, however, CAVDs is a rare condition found in only 1–2% of all infertile men (Bieth et al. 2020).

Optimization and upscaling of Sanger sequencing on automated DNA sequencers resulted in wide-scale adoption of DNA sequencing in human disease research and diagnostics in the early 2000s (Metzker 2005; Hutchison 2007). It was particularly successful in testing for mutations in candidate disease genes identified by either positional cloning or by evidence from orthologous genes studied in model organisms. A good example of this was shown by the identification of mutations in *SYCP3* gene related to meiotic arrest during spermatogenesis (Miyamoto et al. 2003). The role of this gene in male infertility had been previously established in mice where male homozygous null mutants for the *Sycp3* gene were found to be infertile due to massive apoptotic cell loss during spermatogenesis (Yuan et al. 2000). After isolation of the human *SYCP3* gene it was found to have an identical function and similar negative consequences to human fertility if disrupted (Martinez-Garay et al. 2002; Miyamoto et al. 2003). Of note, while in mice bi-allelic mutations are required to reveal an infertility phenotype, in humans a single heterozygous mutation in *SYCP3* is sufficient to compromise spermatogenesis.

## The present: technological advances allowing unbiased analysis of the infertile genome

### Genome-wide homozygosity screening using microsatellite scans and SNP microarrays

Adaptations made to existing sequencing technology as well as the introduction of single nucleotide polymorphism (SNP) microarrays in the 1990s permitted once more a shift in the research approaches available to investigate the genomes of infertile men and identify novel genes associated with male infertility. Initially, this work was focused on developing and applying large sets of polymorphic markers to screen genomes of infertile men from consanguineous descent for regions of homozygosity. In 2007, this new positional cloning approach resulted in the identification of two novel male infertility genes, *AURKC* and *SPATA16*, causing multiple morphological sperm abnormalities (Dieterich et al. 2007; Dam et al. 2007). Similarly, homozygosity mapping was used to identify an homozygous variant in *DNAH1* in a small cohort of infertile men with morphological abnormalities in their sperm flagella (Ben Khelifa et al. 2014).

### Microarray-based detection of genomic copy number variation

While structural variants such as CNVs present on the Y chromosome have been clearly shown to have a negative effect on spermatogenesis (Nathanson et al. 2005; Visser et al. 2009; Krausz and Casamonti 2017), only few other structural genomic variations have been robustly linked to male infertility. Microarray-based comparative genomic hybridization (array CGH) and SNP arrays have dramatically increased the detection resolution of genomic deletions and duplications since the late 90 s (Solinas-Toldo et al. 1997; Pinkel et al. 1998).

The application of SNP microarray technology revealed one important CNV associated with male infertility in 2011, a 200 kb homozygous deletion affecting the *DPY19L2* gene (Harbuz et al. 2011; Koscinski et al. 2011). Specific amplification and sequencing of *DPY19L2* was initially hampered by the presence of a pseudogene. In 2012, Coutton et al*.* optimized the conditions to specifically amplify and sequence this gene and identified globozoospermia patients with a combination of a deletion and a nonsense or missense mutation, as well as a patient with homozygous missense mutations (Coutton et al. 2012). Together, deletions and point mutations in *DPY19L2* are now known to explain most cases of globozoospermia.

In 2011, Tüttelmann et al. employed microarray technology to discover an excess of rare CNVs on the sex chromosomes of azoospermic and severe oligozoospermic German men (Tüttelmann et al. 2011), a signature that was later replicated in other populations (Stouffs et al. 2012; Krausz et al. 2012; Lopes et al. 2013; Lo Giacco et al. 2014). More recently, high-resolution array-CGH was used by Yatsenko et al. to identify an identical deletion of three exons of the *TEX11* gene on chromosome X in two patients with azoospermia. Sanger sequencing of this gene revealed additional pathogenic (truncating and splice) mutations in azoospermia patients (Yatsenko et al. 2015). Mutations in *TEX11* were also reported in azoospermia patients by another group in the same year (Yang et al. 2015). Interestingly, this group decided to study *TEX11* because they previously showed that male mice lacking this gene show meiotic arrest, resulting in azoospermia (Yang et al. 2008).

Using a similar microarray approach, recurrent deletions affecting the *DMRT1* gene located on chromosomes 9 have been associated with azoospermia (Lopes et al. 2013; Lima et al. 2015). While putative pathogenic mutations in azoospermic men have also been described in this gene (Tewes et al. 2014), it’s role in infertility is still not completely clear. The clinical relevance of other rare CNVs, reported in a limited number or even a single infertile man, remains uncertain. As an example, an approximately 1 Mb deletion found in a single azoospermic man was reported in 2014. The CNV on chromosome 11 spans nine genes, of which the *WT1* gene was hypothesized as the potential cause for spermatogenic failure (Seabra et al. 2014). Another example involves an 11–15 kb deletion on chromosome X observed so far exclusively in azoospermic men from the Mediterranean, causing a partial deletion of the proximal copy of the *MAGEA9B* gene (Lo Giacco et al. 2014; Shen et al. 2017).

While SNP and CGH microarrays have highlighted the important role of CNVs in male infertility, the widespread application of next-generation sequencing (NGS) now offers the opportunity to combine the detection of CNVs and other more complex structural variations with the simultaneous detection of SNVs (Zhou et al. 2018; Ho et al. 2019). Recently, a balanced reciprocal translocation affecting the *SYCP2* gene was reported in a patient with severe oligozoospermia, initially discovered by karyotyping but further characterized using both microarrays as well as NGS (Schilit et al. 2020). Interestingly, through international collaboration, loss-of-function mutations affecting this gene were identified in an additional three infertile patients from Germany, highlighting the importance of combining structural genomic variation and single nucleotide variation analysis as well as multi-institutional collaboration.

### Next-generation sequencing to detect new disease genes

**Fig. 2:**
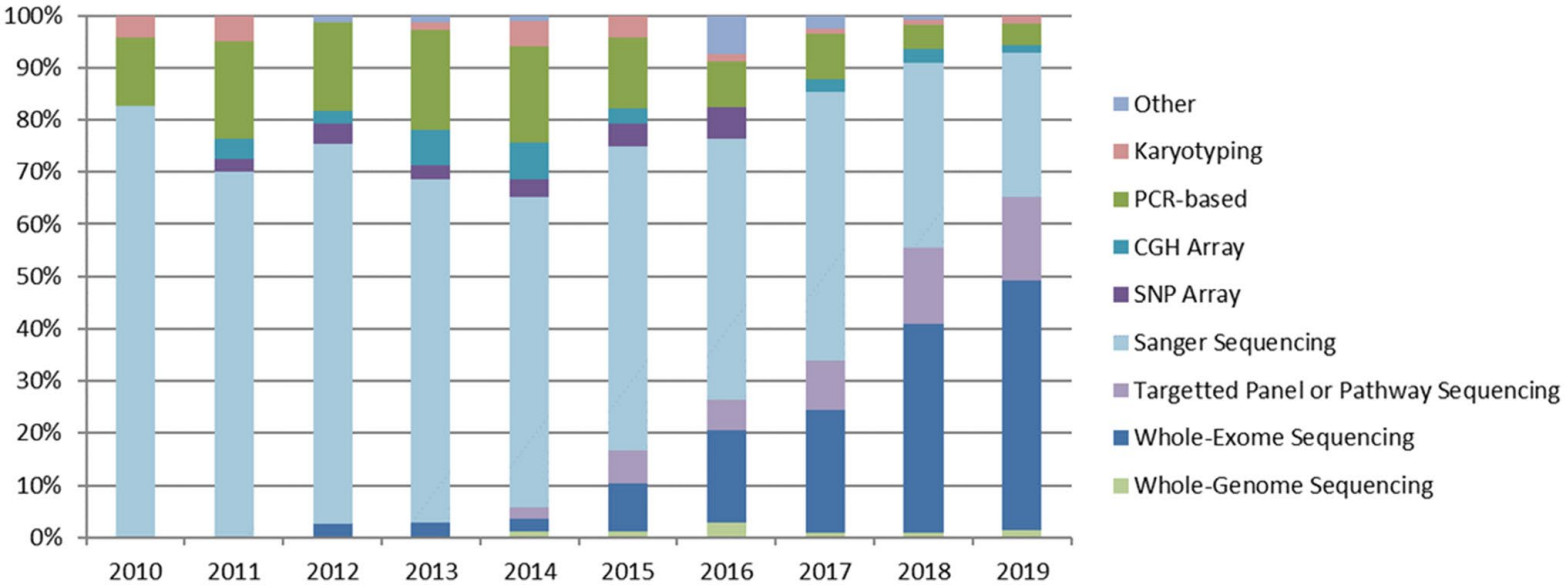
Breakdown of genetic tools used in published male infertility studies over the past decade. Based on an analysis of all published literature related to monogenic forms of male infertility, Sanger sequencing was the predominant tool used (82%) in 2010 but has since then seen a reduced usage down to 26% in 2019. In the same time period, NGS-based techniques gained ground to become currently the most common tool (61%)

The development of high-throughput NGS platforms in the past decade has resulted in a dramatic drop in sequencing costs and an equally dramatic increase in sequencing throughput. Rather than relying on the need to select and sequence individual candidate genes or evaluate specific polymorphisms or large structural defects in a small number of patients and controls, NGS allows for unbiased sequencing of large numbers of genes, all coding exons (whole exome sequencing) or sequencing of the entire human genome, and increasingly allows this to be done in very large cohorts of patients and controls. Accordingly, NGS has provided an inexpensive and rapid genetic screening approach to discover novel disease-associated genes (Boycott et al. 2013; Fernandez-Marmiesse et al. 2018). Figure 2 shows how in recent years exome sequencing has become the predominant technology used for disease gene studies in male infertility.

In Multiple Morphological Abnormalities of the Sperm Flagella (MMAF), exome sequencing of relatively small cohorts of patients has been particularly successful, resulting in the identification of many important new recessive disease genes (Touré et al. 2020). Exome sequencing has also been successfully applied to identify new disease genes for patients with acephalic spermatozoa. In particular, homozygous and compound heterozygous mutations in testis-specific genes *BRDT*, *SUN5* and *PMFBP1* (Zhu et al. 2016, 2018; Li et al. 2017; Sha et al. 2019) have been found to disrupt the head-flagella junction of the spermatozoa of these infertile men. In addition, NGS has also been instrumental in the study of congenital isolated hypogonadotropic hypogonadism, characterised by incomplete or absent puberty and infertility. A large number of pathogenic mutations have been identified in genes and genetic loci that result in neurodevelopmental defects of gonadotropic hormone-releasing hormone (GnRH) neuron migration or disrupt neuroendocrine GnRH secretion and action (Cangiano et al. 2020; Butz et al. 2020).

The investigation of the genetic basis for the much more common forms of non-obstructive azoospermia has proven to be more difficult, likely because of the enormous genetic heterogeneity resulting in a wide diversity of phenotypes encompassed in NOA, from the complete lack of germ cells (Sertoli cell only syndrome) to various forms of maturation arrest. Nevertheless, significant progress has been made on this front, where exome sequencing of azoospermic men in both consanguineous and non-consanguineous families has revealed pathogenic mutations in genes such as *TEX15* (Okutman et al. 2015)*, FANCM* (Kasak et al. 2018)*, XRCC2* (Yang et al. 2018)*, MEIOB* (Gershoni et al. 2019) and *FANCA* (Krausz et al. 2019). In addition, exome sequencing revealed mutations in *ADGRG2* in patients with congenital obstructive azoospermia (Patat et al. 2016). In conclusion, the widespread application of exome sequencing in azoospermia and severe oligozoospermia is uncovering mutations in many new candidate disease genes (see also Kasak and Laan 2020). At this stage, however, many of these new candidate genes have not been independently replicated and their role in quantitative sperm defects therefore remains to be established.

### Clinical validity assessment of candidate infertility genes

Up to this point we have described the different approaches used to identify genetic variants associated with male infertility. However, in order to convincingly link variants in a certain gene to disease, one needs to consider various levels of evidence. Incorrect and misleading conclusions about the role of genes in causing quantitative or qualitative spermatogenic defects could lead to inappropriate diagnoses and even mismanagement and counselling of couples. Furthermore, incorrectly characterized genes may impede follow-up research by contaminating candidate disease gene lists and pathway analyses. It is therefore important to identify genes with insufficient, inconclusive and low-quality evidence for involvement in the aetiology of male infertility. Recently, a systematic clinical validity assessment of all evidence available for gene-disease relationships in male infertility was performed (Oud et al. 2019). Based on a previously published method (Smith et al. 2017), 92 genes were classified as at least moderately linked to a human male infertility phenotype. This included only 18% of all 521 gene-disease relationships described in male infertility at that time, demonstrating that the quality and extent of evidence varies greatly.

In the years to come, the number of novel genes described for male infertility is expected to increase rapidly as NGS methods become even more widely and frequently used. It is therefore important to continue adding and regularly re-assessing the genetic and functional evidence for all genes described (Soraggi et al. 2020; Houston et al. 2020).

### Recent developments in functional validation

**Fig. 3:**
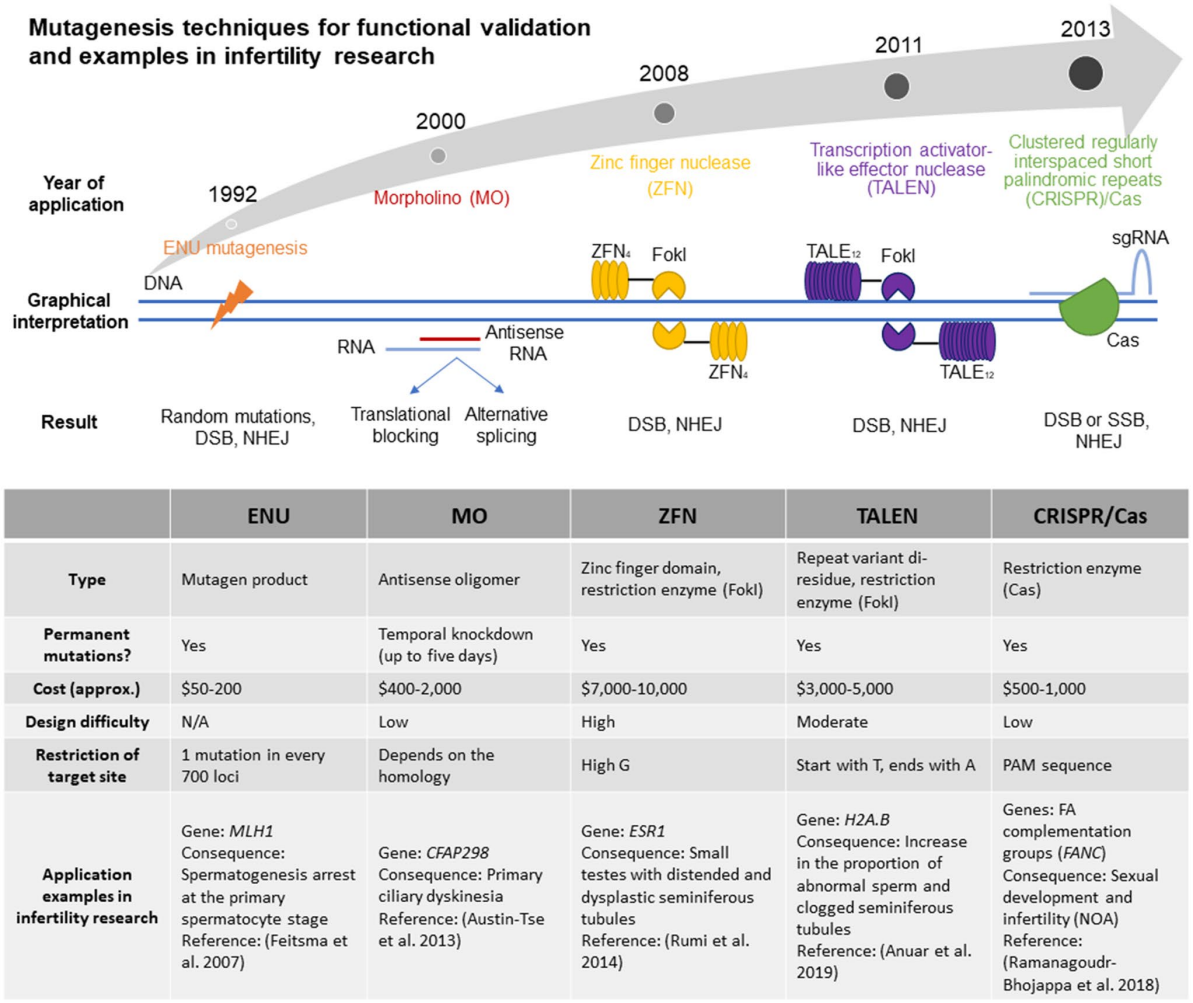
Mutagenesis techniques for functional validation and examples in male infertility research**.** Outline of the current mutagenesis techniques for functional validation, and the approximate year of application. Comparison of the main characteristics of the described techniques and some published examples of the application of these technologies in male infertility research

The proper design of functional validation experiments, as well as reliance on previously developed functional models with male infertility phenotypes, are critical for the validation of newly identified variants. The emergence of new techniques for gene editing and the implementation of novel model organism and in vitro systems for the study of spermatogenesis has and will continue to expand our ability to perform the critical step of functional validation (Fig. 3).

In many diseases, in vitro modelling systems are widely used and highly effective in screening large numbers of variants across multiple genes. Additionally, in many cases in vitro systems can be established from individuals carrying a specific variant, enabling the direct assessment of the functional impact of the variant. However, despite significant efforts, the development of in vitro spermatogenesis systems in humans has proven elusive to date (Komeya et al. 2018). The ability to culture and maintain the function of human testicular tissue would enable the functional validation of novel variants, and perhaps the modification of variants that cause azoospermia, potentially enabling the restoration of spermatogenic capacity in the not-too-distant future (Sato et al. 2011; Ibtisham et al. 2017).

While in vitro systems are not well established for the study of male infertility, the application of gene editing tools in a variety of model organisms has proven effective in the functional validation of suspected infertility-causing variants. For example, in the mouse, *N*-ethyl-*N*-nitrosourea (ENU), an alkylating agent that induces genome-wide mutagenesis at random loci (Lewis et al. 1992; Stainier et al. 2017), has been successfully used for the identification of novel genes required for male fertility. Following random mutagenesis by ENU, mice displaying an infertility phenotype are subsequently screened by a variety of approaches, including selective breeding and linkage analysis, and more recently whole genome sequencing, to identify the mutations underlying reproductive defects (Kennedy and O’Bryan 2006; Jamsai and O’Bryan 2010; Geister et al. 2018).

In cases where a candidate variant has been identified and requires functional validation, a variety of targeted approaches can nowadays be applied. These approaches include the use of morpholino oligonucleotides for relatively short-term suppression of gene activity, zinc finger nucleases (ZFN), transcription activator-like effector nucleases (TALEN) and Clustered regularly interspaced short palindromic repeats (CRISPR) (Summerton 1999; Gaj et al. 2013; Rumi et al. 2014; Ramanagoudr-Bhojappa et al. 2018; Anuar et al. 2019), which enable genomic modification to inactivate a gene through the introduction of a premature stop codon or frameshift mutation. Alternatively, these approaches can be used to recapitulate specific mutations, for example a missense mutation that is predicted to be pathogenic (Gaj et al. 2013; Anzalone et al. 2019). While all of these tools have been applied successfully in male infertility research, the advantages of CRISPR in terms of cost, efficiency and simplicity have made this gene editing technology the tool of choice for most contemporary studies, and its use in male infertility research will certainly expand rapidly in the coming years.

The enormous value of mouse knock-out experiments for the identification of human male infertility genes has already been highlighted in early studies (Lyon and Hawkes 1970; Yuan et al. 2000). For example, studies of the murine *Klhl10* gene, shown to be essential for spermiogenesis in mice, motivated male infertility studies in humans and pointed to one of the few known examples of autosomal dominant male infertility (Yan et al. 2004). After employing reverse-transcription PCR on RNA isolated from sperm of infertile and healthy men, two pathogenic-predicted *KLHL10* missense mutations and one splicing mutation were identified by Sanger sequencing in severely oligozoospermic patients. Of note one fertile man was found to harbour one of these missense mutations, questioning the penetrance of this variant (Yatsenko et al. 2006). Another example is *hsf2,* which was initially found to regulate normal spermatogenesis in mice (He et al. 2003) and it was only later that dominant negative heterozygous mutations in the human orthologue *HSF2* were found in infertile men by targeted NGS sequencing of this and ~ 600 other infertility candidate genes (Mou et al. 2013).

Zebrafish have been used increasingly to model human diseases including infertility. Lin et al*.* showed using a zebrafish model and Crispr/Cas9 that the *amh* gene is essential to control the balance between proliferation and differentiation of male germ cells (Lin et al. 2017). More recently CRISPR technology was used to systematically characterize the function of 17 Fanconi Anemia (FA) genes, revealing their essential role in growth, sexual development and fertility (Ramanagoudr-Bhojappa et al. 2018). Studies like these are essential for the clinical validity assessment of genes related to male infertility and provide additional evidence for the role of recently identified mutations of these genes in azoospermic men (Kasak et al. 2018; Krausz et al. 2019).

High throughput screens in Drosophila using ethyl-methanesulfonate-induced mutations, similar to the ENU approach in mice (Wakimoto et al. 2004) or more recently RNA interference (RNAi) knockdown (Yu et al. 2015) enabled the efficient identification of candidate infertility genes and downstream functional assessment of multiple candidate genes in a single experiment. For example, discovery of the role of *boule* (homologous to human *DAZ*) in meiosis was first discovered in a large-scale mutation screen using drosophila (Castrillon et al. 1993; Eberhart et al. 1996).

Lastly, flagellated and ciliated unicellular organisms including Chlamydomonas, Trypanosoma and Tetrahymena have been used to study the functional consequences of mutations in genes involved in sperm flagellar formation and function (e.g. *CFAP43, CFAP44, ODA7, SPAG16L*, and *WDR66*) in patients displaying MMAF (Zhang et al. 2006; Duquesnoy et al. 2009; Coutton et al. 2018; Kherraf et al. 2018). Yeast have likewise been an important model organism for the study of meiosis, enabling the identification of novel meiotic genes associated with male infertility, for example *gda1* -orthologous to human *ENTPD6* (Wang et al. 2015).

The use of gene editing tools in these and other model organisms will continue to be important in identifying and characterizing genetic variants associated with male infertility.

## The future: overcoming existing limitations to improve biological understanding, patient diagnostics and prognostics

The introduction of unbiased NGS approaches have revolutionized the identification of genetic causes for many diseases (Payne et al. 2018; Bean et al. 2020)*.* Although these methods are frequently used in research laboratories to study the genetics of male infertility, they have yet to identify the majority of genes underlying this disorder. For several types of male infertility such as DFS-MMAF, acephalic sperm syndrome and globozoospermia, the current diagnostic yield based on known genetic causes explains approximately 50% of cases. In contrast, the diagnostic yield for the most common forms of male infertility, azoospermia and oligozoospermia, remains much more limited (Krausz 2011; Tüttelmann et al. 2018; Krausz and Riera-Escamilla 2018). Given the enormous potential of new genomic technologies in both research and diagnostics, how can we most efficiently improve our understanding of the genetics of male infertility? To conclude this review, we will discuss the limitations and pitfalls of current approaches and highlight some important new developments in genomic technologies and improvements in the organization of our research that should help to accelerate disease gene identification.

### Challenges to gene discovery in a relatively common heterogeneous disorder

Exome and especially genome sequencing now allow us to perform unbiased genetic studies that can help to identify novel disease genes or genomic regions. The power to discover novel infertility genes is, however, still heavily influenced by a number of study design considerations. As with other disease models, careful phenotyping and cohort selection are critical. In addition, an understanding of the genetic architecture of the disease is important in designing appropriately powered studies. As previously discussed, discrete sperm morphological abnormalities are expected to involve a relatively modest number of genes, whereas a quantitative spermatogenic failure phenotype may arise from variants in any one of hundreds or even thousands of genes. In the case of extremely rare disorders with discrete phenotypes, a small case/control design may be appropriate, as it is likely that variants will be localized to a small number of genes. Genetic studies in consanguineous families have proven to be an effective means of identifying novel male infertility genes accounting for rare qualitative sperm defects. In cases of consanguinity, analyses can be focussed on regions of the genome that are homozygous by descent, as it is likely that rare recessive disease-causing mutations will exist in a homozygous state more frequently in these families (Okutman et al. 2015; Kasak et al. 2018).

In the investigation of NOA in outbred populations, it is likely that individual genes will harbour disease-causing variants at extremely low frequency in the patient population, given the effect of the disease on overall fitness. In this case, large patient and control cohorts and robust statistical methods are essential to identify genes with an increased mutational load in the genome of affected patients, as was shown for other genetically heterogeneous disorders (Fitzgerald et al. 2015; Wilfert et al. 2016). This calls for large-scale collaborative studies and widespread data sharing to reliably and robustly identify and functionally validate new male infertility genes.

International consortia have recently been established in the field of male infertility genetics to promote this, including the GEMINI consortium (https://gemini.conradlab.org/) and the IMIGC consortium (https://www.imigc.org/). Collaboration, both within and beyond these consortia, will be essential to identify recurrently mutated genes, study detailed clinical presentations, perform relevant functional studies to confirm the pathogenicity of certain mutations and unravel the underlying biological mechanisms. Moreover, these consortia can help to replicate single case observations and confirm or question the role of new candidate genes in infertility, establish clinical guidelines and help to improve genetic diagnostics. Increased research collaboration can be a driver that benefits both our biological understanding as well as patient diagnostics in the field of male infertility.

### Establishing the role of inherited and de novo mutations in infertility

So far, most research has focused on studying either autosomal recessive or X/Y-linked forms of male infertility. Severe male infertility cannot be dominantly inherited from fathers, but it can be maternally inherited or caused by de novo germline and post-zygotic mutations or CNVs. Exome and genome sequencing of patient-parent trios has revealed the importance of de novo germline mutations in many sporadic genetic diseases, in particular in intellectual disability and related disorders (Vissers et al. 2010, 2016; Veltman and Brunner 2012; McRae et al. 2017). This approach can be an immensely powerful means of identifying novel disease genes, but one of the main challenges for applications in male infertility research is that parental DNA samples are often unavailable. Recently, however, several groups have started to assemble cohorts of patient-parent trios for male infertility genetic research, aiming to identify de novo mutations causing male infertility as well as providing insight into dominant maternal inheritance.

### Towards a comprehensive overview of all genomic variation in male infertility

Most NGS approaches use short-read sequencing which limits the detection of repeat structures (Schatz et al. 2010), highly homologous sequences and structural variation (Zhou et al. 2018; Ho et al. 2019). This affects male infertility research significantly, given the importance of chromosome microdeletions in highly homologous and repetitive regions on the Y chromosome as well as causal structural variation reported in other genomic regions. Recently, new sequencing platforms have emerged that have increased sequencing read lengths from a few hundred nucleotides to 10 kb or even longer (Rhoads and Au 2015; Gordon et al. 2016; Jiao et al. 2017). Because of their increased read length these platforms are much better able to detect repeat expansions, homologous sequences and structural genomic variation (Pendleton et al. 2015; Cretu Stancu et al. 2017). Unfortunately, the current per-base accuracy and cost of these technologies does not yet compare favourably to short-read sequencing methods (Rhoads and Au 2015; Rang et al. 2018). Therefore, it is not yet possible to reliably and affordably ascertain all genetic variation present in the genome of an infertile patient in a single experiment. However, complete, accurate and affordable human genome sequencing will likely be available in the next decade, and the major challenge in male infertility will become variant interpretation, not detection.

## Conclusion

Significant progress has been made in our understanding of the genetics of male infertility. Using a variety of genomic technologies, the genes involved in rare qualitative sperm defects have now been largely identified, resulting in the development and application of NGS gene panels with a high diagnostic yield. Progress in our understanding of the genetics underlying the more common quantitative sperm defects has not been as rapid as we have seen in qualitative sperm defects or in other genetic disorders. The organization of large well-funded consortia studying thousands of patients with unbiased genomics approaches and the application of relevant functional validation assays should result in a much-needed breakthrough in this field in the coming years.
